# Single-port robotic surgery for mediastinal tumors using the da vinci SP system: Initial experience

**DOI:** 10.3389/fsurg.2022.1043374

**Published:** 2022-10-31

**Authors:** Bo Yang, Ruiji Chen, Yingxue Lin, Yang Liu

**Affiliations:** ^1^ Department of Thoracic Surgery, First Medical Center, Chinese General Hospital of PLA, Beijing, China; ^2^ Department of Thoracic Surgery, Hainan Hospital of Chinese General Hospital of PLA, Sanya, China; ^3^ School of Medicine, Nankai University, Tianjin, China

**Keywords:** single-port robotic surgery, mediastinal tumors, da vinci SP system, minimally invasive thoracic surgery, superior mediastinal tumors

## Abstract

**Purpose:**

Studies of single-port robot-assisted thoracic surgery (RATS) using the da Vinci SP system, which uses a smaller surgical incision than the conventional multiport robot, have yet to be reported because of its smaller operating range. We report our initial experience using the da Vinci SP system in thoracic surgery for the resection of mediastinal tumors that requires a smaller workspace.

**Description:**

Two patients diagnosed with superior mediastinal tumors underwent RATS performed with the da Vinci SP surgical system in January 2022. We used three-dimensional reconstruction to preoperatively determine the surgical incision. This is the first report of single-port RATS using the SP system in China.

**Evaluation:**

R0 resection was achieved in both operations without complications. Operation times and bleeding volumes were similar to the use of multiport RATS. No perioperative complications occurred.

**Conclusions:**

The da Vinci SP system can be used for the resection of superior mediastinal tumors. Case selection and preoperative planning should be performed prior to these surgeries.

## Technology

Single-port robot-assisted thoracic surgery (RATS) using the da Vinci SP system has yet to be reported. Robot-assisted surgery has demonstrated superiority in the resection of mediastinal tumors, particularly tumors in the superior mediastinum (1). In the past 5 years, our group has performed more than 40 consecutive robot-assisted surgeries for superior mediastinal tumors and accumulated valuable technical experience. In January 2022, two cases of single-port robot-assisted surgery for superior mediastinal masses were performed in our institute using the da Vinci SP system. To our knowledge, this was the first use of the da Vinci SP for single-port thoracic surgery in China.

## Technique

First, we performed a three-dimensional (3D) reconstruction to choose the incision in case all workspace was covered in the SP system operating range. Then, SP RATS was performed according to preoperative planning. Patients' characteristics and perioperative data were recorded to evaluate technical feasibility.

## Clinical experience

### Material and methods

#### Patients

**Figure 1:**
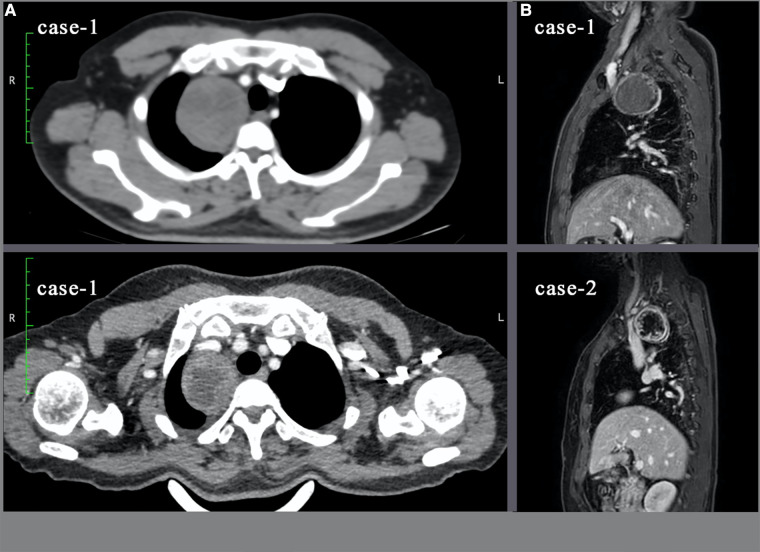
Ct and MRI images of both tumors. (**A**): Cross-sectional view of the tumor on CT examination; (**B**): Sagittal view of MRI. In case-2, the tumor has cystic component.

Two consecutive patients diagnosed with superior mediastinal tumors underwent RATS performed with the da Vinci SP surgical system in January 2022. Case 1 was a 48-year-old female with an incidental finding of a mediastinal mass during a medical checkup without clinical symptoms. Case 2 was a 45-year-old male presenting with right ptosis and blurred vision who was diagnosed with Horner's syndrome. A mediastinal mass was found on subsequent CT examination (Figure 1A). Both surgeries were performed by the same surgeon who had performed more than 40 surgeries for mediastinal tumors using the conventional da Vinci Si or S robot over the last 5 years and had completed da Vinci SP system training and certification.

#### Preoperative procedure

**Figure 2:**
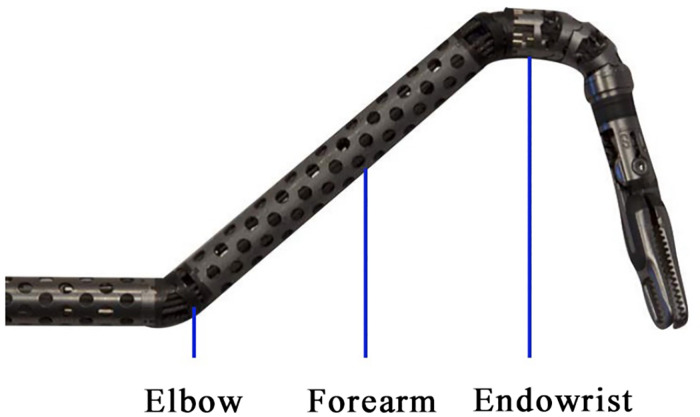
Demonstration of the flexible double-jointed instrument. The movement of the “elbow” joint ensures that the lens does not conflict with the two Endowrists.

**Figure 3:**
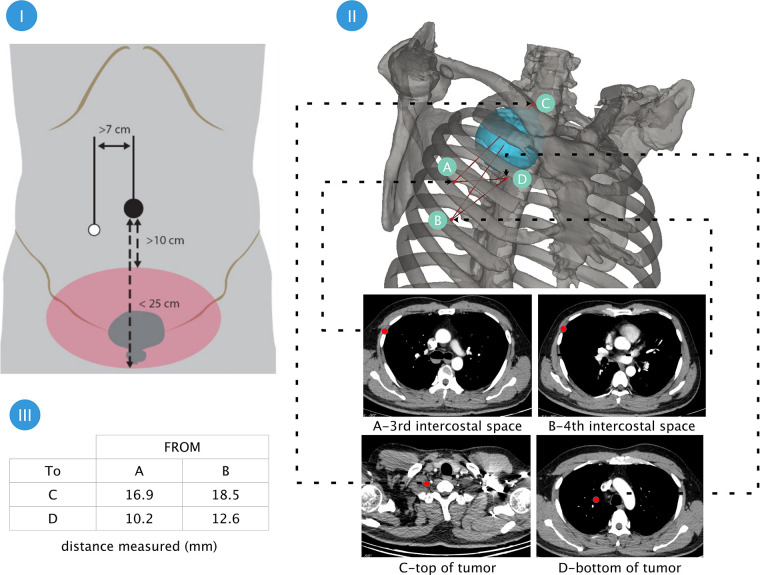
Planning the surgical incision and simulation of the workspace through preoperative 3D reconstruction ensured the surgical area be covered. Part I:According to the instructions, the cannula should be positioned at a distance from the operating area 10 cm–25 cm; Part 2: 3D reconstruction using OsiriX software (Fondation OsiriX, Geneva, Switzerland) and measurement of the distance between the 3rd and 4th intercostal spaces to the uppermost and lowermost Poles of the tumor. Part III:display of measurement results.

Both patients underwent an MRI examination to exclude mass extension through the intervertebral foramen and vascular or nerve invasion (Figure 1B). Preoperative cardiopulmonary function and other basic assessments were favorable, with no contraindications to general anesthesia observed. As the instrument arm position was limited, the surgical procedure relied entirely on the activity of the “elbow” and multijoint “endowrist” (Figure 2). 3D reconstruction was performed preoperatively to determine the incision location using OsiriX software (Fondation OsiriX, Geneva, Switzerland), which can be downloaded free from the Internet. The choice of incision was based on two principles. According to the SP system instructions, the location for the SP cannula should be greater than 10 cm from the nearest border of the surgical workspace and less than 25 cm from the farthest border of the surgical workspace. Then, the cannula and the center of the tumor should be maintained in a straight line. We made measurements based on the above criteria (Figure 3). Incisions were selected to allow full instrument articulation and reach.

#### Surgical procedure

**Figure 4:**
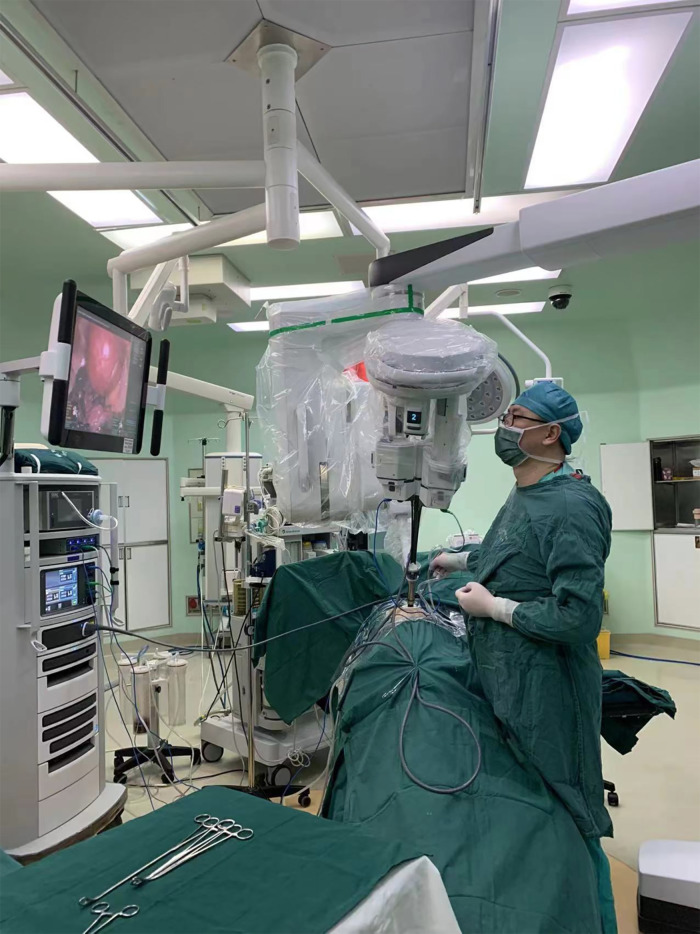
Robot docked over the head.

**Figure 5:**
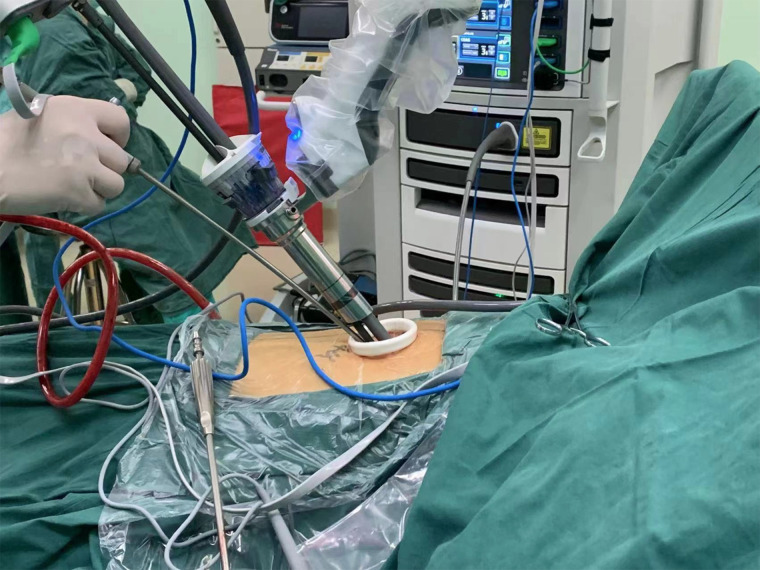
Placement of the instruments and suction apparatus used by bedside assistant.

Both patients were positioned in the lateral decubitus position after general anesthesia with the use of a double-lumen endotracheal tube (Covidien IIc, Athlone, Ireland). According to preoperative 3D reconstructions, a 4 cm incision was made in the third (case 1) and fourth (case 2) intercostal space around the anterior axillary line. An incision retractor (HK-60/70-60/100) was used to enlarge the intercostal space. The robot was then docked over the head (Figure 4). The diameter of the cannula used with the SP system was 25 mm. Because of the limited intercostal width, the cannula was unable to be directly inserted into the intercostal space. Therefore, we mounted the cannula outside the body to ensure alignment between the incision and the tumor. A 3D camera lens and operating instruments were passed through the cannula and the intercostal space into the thoracic cavity. We favored using the camera at the 12 o'clock position with a permanent cautery hook (Surgical Intuitive, Mountain View, CA, USA) at 3 o’clock (arm 2) and fenestrated bipolar forceps (Surgical Intuitive, Mountain View, CA, USA) at 9 o'clock (arm 1). The instruments of arms 1 and 2 were interchanged when required. The bedside assistant used suction apparatus to assist the operation through the same incision site (Figure 5).

**Figure 6:**
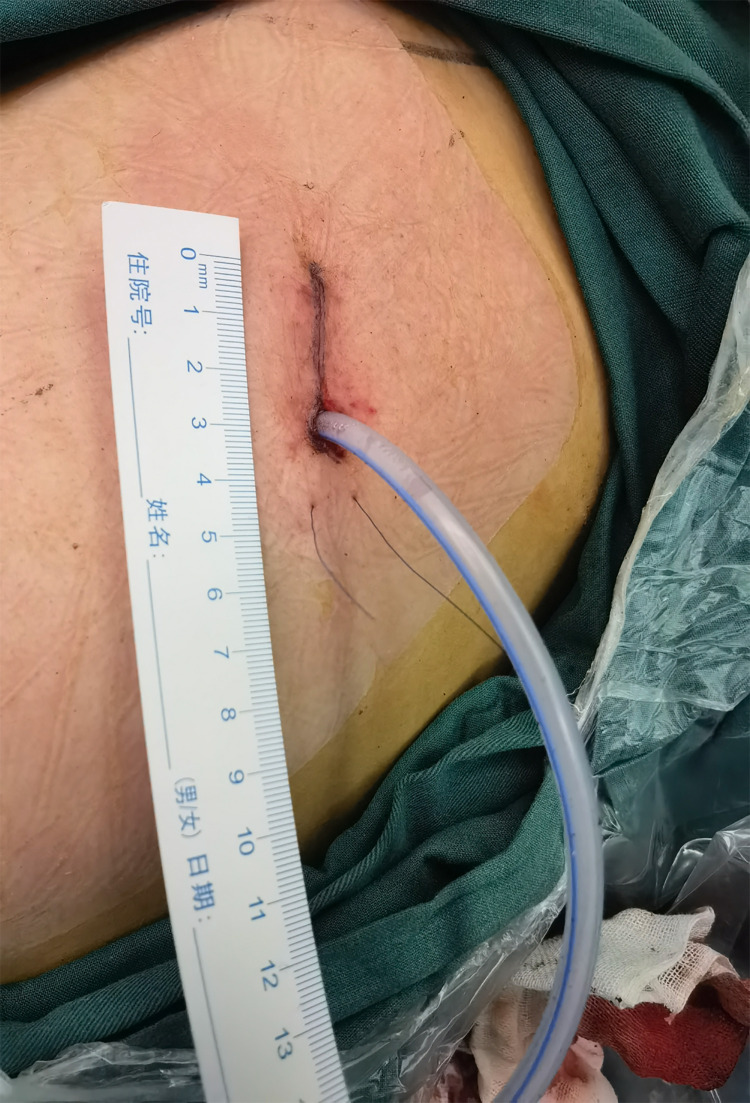
Placement of drainage tube, incision length and cosmetic result after suture.

First, the relationship between the tumors and the sympathetic nerve chain was explored. Both tumors were found to be outside the pleura. The tumor in case 2 was found to originate from the pleura. Consistent with preoperative imaging, both tumors had intact envelopes and no clear trophoblastic vessels. Tumors were separated along their borders. Extreme care was taken to prevent damage to subclavian vessels. The use of electrical energy devices was avoided near sympathetic nerves. Based on MRI findings, the tumor capsule in case 2 was incised to internally decompress the cyst to increase the operative field. Both tumors were removed through the incision with a sample bag after complete resection. A 16-Fr drainage tube was placed through the same incision site as per routine practice (Figure 6).

## Results

A 3D view of the surgical field allowing full exposure and separation was provided using the fully wristed camera and double-jointed instruments. Pathological examination demonstrated schwannoma in both cases. R0 resection was achieved in both cases. Conversion to video-assisted or open surgery was not required in either case. Table 1 shows the patients' demographics and perioperative data. The total operative time was 113 and 103 min, respectively. No intraoperative complications occurred, with intraoperative blood loss volumes of 50 and 100 ml, respectively. These results are comparable with the use of conventional multiport RATS. No complications greater than Clavien–Dindo (2) grade I occurred postoperatively. Drainage tubes were removed in both patients on the first postoperative day when the following criteria were met: plain chest radiography demonstrating no pneumothorax, pleural effusion, or focal consolidation; drainage volume less than 100 ml per day; and no obvious abnormality in laboratory measures, including routine blood tests, inflammatory markers, and indicators of coagulation function. Both patients were discharged on the second postoperative day.

**Table 1:** Patients’ characteristics and perioperative data.

Characteristic	Patient NO. 1	Patient NO. 2
Age (year)	48	45
Sex	Female	Male
Body mass index (kg/m^2^)	23.83	27.46
Operative time (min)	113	103
Docking time (min)	15	10
Console time (min)	86	90
Suture time (min)	12	13
Intraoperative complications	No	No
Conversion to other surgery	No	No
Estimated blood loss (ml)	20	100
Tumor size (cm)	5 × 5 × 3	7 × 5 × 2.5
Histological type	Schwannoma	Schwannoma
Duration of the chest tube	1	1
Total chest tube drainage	100	40
Discharge	POD 2*	POD 2*
Pain at discharge,VAS score	3	5
Postoperative complications	No	No

*POD: postoperative day.

Outpatient follow-up was conducted 1 month postoperatively. Both patients recovered well. There was no obvious amelioration of Horner's syndrome (right ptosis and blurred vision) in case 2. Satisfactory cosmetic results were achieved in both patients.

## Comment

The da Vinci SP robot, which represents a more minimally invasive surgical approach, has been successfully used in urology (3–5) and gynecology (6) surgeries. Although conventional multiport robot-assisted surgery has long been used for the treatment of various thoracic diseases, da Vinci SP RATS has not previously been reported because of its limited workspace and the large operating range required for thoracic surgery. First, the operating depth range was only 15 cm. As the diameter of the cannula was much larger than the intercostal space, we referred to previous reports of single-port transoral robot-assisted surgery (7) and left the cannula outside the thoracic cavity while inserting the camera and instruments into the cavity. This approach solved the docking problem but further shortened the operating range. Second, movements in both up/down and left/right directions rely on the rotation of the instrument arm, which could not be performed because of the limitations of the thoracic bony structure, particularly when the required movement was in the direction vertical to the incision. Neurogenic tumors in the mediastinum required only a limited surgical range, which can be met by the SP robot despite the above limitations. Previous experience with multiport RATS for mediastinal tumors has shown that the location of the incision should be individualized (8). This is particularly important in SP RATS. Based on our experience in planning pulmonary segmentectomy using 3D reconstruction (9), we believe that 3D reconstruction can accurately measure the operational limits that may be encountered intraoperatively. Planning the surgical incision and simulation of the workspace through preoperative 3D reconstruction (10) ensured that the surgical area could be covered. As a next step, we aim to explore the use of 3D printed models to simulate surgical incisions. The surgical procedure was performed without the use of artificial pneumothorax, and the bedside assistant was able to use the incision for additional retraction and suction as well as specimen retrieval, which represents an advantage of the SP robot.

We believe that these two successful surgeries demonstrate that SP robot-assisted surgery can be successfully used to perform resection of small- to medium-sized mediastinal tumors. The SP robot is advantageous for surgery with limited surgical space, such as the resection of esophageal smooth muscle tumors and neurogenic tumors. However, this approach is not feasible for surgeries requiring wide operative fields such as the resection of esophageal and lung cancers. Accordingly, further studies of surgical methods are required before the SP robot can be applied to these surgeries. Additionally, we were unable to compare the advantages and disadvantages of the SP robot compared with the previous generation of multiport robots because of the small sample of the present study.

To our knowledge, this is the first report of single-port RATS using the da Vinci SP robot system in China. We plan to extend the findings of the present study to evaluate the utility of this system in lung and esophageal surgeries.

## Disclosures and freedom of investigation

The da Vinci SP system used in this study was purchased by our institute. The authors are fully responsible for the design of the study, methods used, outcome measurements, analysis of data, and production of the written report.

## Data Availability

The original contributions presented in the study are included in the article/Supplementary Material, further inquiries can be directed to the corresponding author/s.

## References

[1] Kang CH Bok JS Lee NR Kim YT Lee SH Lim C. Current trend of robotic thoracic and cardiovascular surgeries in Korea: analysis of seven-year national data. Korean J Thorac Cardiovasc Surg. (2015) 48(5):311–7. 10.5090/kjtcs.2015.48.5.311 26509124 PMC4622026

[2] Dindo D Demartines N Clavien PA. Classification of surgical complications: a new proposal with evaluation in a cohort of 6336 patients and results of a survey. Ann Surg. (2004) 240(2):205–13. 10.1097/01.sla.0000133083.54934.ae 15273542 PMC1360123

[3] Agarwal DK Sharma V Toussi A Viers BR Tollefson MK Gettman MT Frank I Initial experience with da vinci single-port robot-assisted radical prostatectomies-ScienceDirect. Eur Urol. (2020) 77(3):373–9. 10.1016/j.eururo.2019.04.001 31010600

[4] Francavilla S Abern MR Dobbs RW Vigneswaran HT Talamini S Antonelli A Single-Port robot assisted partial nephrectomy: initial experience and technique with the da vinci single-port platform (IDEAL phase 1). Minerva Urol Nephrol. (2022) 74:216–24. 10.23736/S2724-6051.21.03919-9 33769009

[5] Kaouk JH Bertolo R. Single-site robotic platform in clinical practice: first cases in the USA. Minerva Urol Nefrol. (2019) 71:294–8. 10.23736/S0393-2249.19.03384-8 30700085

[6] Heo JE Kang SK Koh DH Na JC Lee YS Han WK Pure single-site robot-assisted pyeloplasty with the da vinci SP surgical system: initial experience. Investig Clin Urol. (2019) 60(4):326. 10.4111/icu.2019.60.4.326 31294143 PMC6607072

[7] Mendelsohn AH Lawson G. Single-port transoral robotic surgery hypopharyngectomy. Head Neck. (2021) 43(10):3234–7. 10.1002/hed.26794 34156733

[8] Kajiwara N Kakihana M Kawate N Ikeda N Appropriate set-up of the da vinci surgical system in relation to the location of anterior and middle mediastinal tumors. Interact Cardiovasc Thorac Surg. (2011) 2:112–6. 10.1510/icvts.2010.251652 21081552

[9] Li C Zheng B Yu Q Yang B Liang C Liu Y Augmented reality and 3-dimensional printing technologies for guiding Complex thoracoscopic surgery. Ann Thorac Surg. (2021) 112(5):1624–31. 10.1016/j.athoracsur.2020.10.037 33275930

[10] Wen J Hou X Chu X Xue X Xue Z Application of three dimensional reconstruction technique in selection of incision of thoracic surgical operation with robot. Int J Clin Exp Med. (2015) 8(10):17818–23. PMID: 26770374; PMCID: PMC4694274 26770374 PMC4694274

